# Novel electromagnetic induction heat curing process of fly ash geopolymer using waste iron powder as a conductive material

**DOI:** 10.1038/s41598-022-13392-x

**Published:** 2022-06-09

**Authors:** Toon Nongnuang, Peerapong Jitsangiam, Ubolluk Rattanasak, Prinya Chindaprasirt

**Affiliations:** 1 Department of Civil Engineering, Faculty of Engineering, Chiang Mai University, Huay Kaew Road, Muang, Chiang Mai, 50200 Thailand; 2 Center of Excellence in Natural Disaster Management, Department of Civil Engineering, Faculty of Engineering, Chiang Mai University, Chiang Mai, 50200 Thailand; 3 Department of Chemistry, Faculty of Science, Burapha University, Chonburi, 20131 Thailand; 4 Sustainable Infrastructure Research and Development Center, Department of Civil Engineering, Faculty of Engineering, Khon Kaen University, Khon Kaen, 40002 Thailand; 5 Academy of Science, Royal Society of Thailand, Dusit, 10300 Bangkok Thailand

**Keywords:** Materials science, Mathematics and computing

## Abstract

Geopolymer (GP) was invented to replace concrete, but its heat curing requirement hinders extensive use in real-world construction. Past studies have tested several methods of heat curing. However, the conventional heat curing process (using an oven) is still required for GP to develop good strength on the laboratory scale. This study introduces a new heat curing method for GP based on an electromagnetic field (EMF)generator and a ferromagnetic material. Waste iron powder (WIP) was used as the ferromagnetic material mixed with the fly ash-based GP to generate heat through induction. The sample was cured at 1.18 kW with 150–200 kHz of EMF generator for 15 min. The results showed that 5% of the WIP mixed sample gained compressive and flexural strength at 28 days more than the control (oven-cured). Compressive and flexural strengths of 76.8 MPa and 11.3 MPa were obtained, respectively. In addition, heat induction enhanced the densification and geopolymerization in the GP matrix following SEM and XRD results. This alternative method of heat curing accelerated the formation of the GP matrix, reduced curing time, and increased strength. Moreover, this EMF curing method can save 99.70% of the energy consumed compared to the conventional heat curing method.

## Introduction

Due to the high emissions of carbon dioxide (CO_2_) from cement clinker production and construction activities, massive investment in green materials is urgently needed to solve this greenhouse effect problem. These efforts are in response to the three achievements of the UN Sustainable Development Goals (SDGs): sustainable cities and communities, responsible consumption and production, and climate action. The climate change negatively affects the way of construction^1^. Hence, waste and by-product materials have received a large amount of attention as alternative materials for construction around the world^2–5^. Using waste is environmentally friendly and reduces the cost of construction^6–8^. One popular and well-known material effectively produced from waste materials such as coal fly ash (FA) is geopolymer (GP). For partial or full concrete replacement, GP has been studied for decades as a substitute for ordinary Portland cement (OPC) concrete^2,9^. This environmentally friendly cementitious material results from the synthesis of an inorganic polycondensation reaction when aluminosilicate mixes with an alkaline solution, discovered by Joseph Davidovits in the 1970s^10,11^. FA is a pozzolanic material widely used to manufacture a GP^12–17^. Due to the endothermic reaction, GP requires a heat curing process at low temperature to attain high strength and good physical properties^18^. A temperature range of 50–80 °C with a curing duration of 24 h or more was suggested^19,20^. However, the heat curing process and long curing period constrain the use of GP in actual construction. Although many researchers have studied alternate forms of heat curing, such as self-curing^21^, room temperature curing^22,23^, hot weather curing^24^, or even microwave radiation^25–28^, the strength of the resulting material is not satisfactory. Researchers have studied advanced curing methods such as hot and cold pressing to obtain ultra-high mechanical properties^29,30^. The short-time curing method is a significant step to increasing geopolymer application in actual construction. For the conventional heat curing technique, which generally requires a hot air oven, the heat energy penetrates through the surface of the material and transfers to the inside of the sample by thermal convection. Nonetheless, due to the gentle thermal gradients inside the material, a slow rate of geopolymerization occurs, leading to incomplete formation of the geopolymeric structure^31^.

A better way of heat curing must be found to apply GP to the pressing need for modern construction materials. Every year, industrial sectors produce many by-products and a large amount of waste. One waste product relevant to the GP problem is waste iron powder (WIP). In Thailand, approximately 20,000 tons of WIP are produced annually, and most of this waste is disposed of by dumping it in pit holes^32^. This disposal method could lead to long-term environmental problems. It has been revealed that this waste iron powder has been utilized with an electromagnetic field (EMF) generator to generate heat by hysteresis loss. Hysteresis loss is caused due to molecular friction in a ferromagnetic material (iron) under an alternating magnetic field. The loss is released as heat energy. Some recent works have applied the metal scraps in asphalt concrete and used an electromagnetic field to induce heat and create a self-healing asphalt concrete^33^. In addition, some researchers have incorporated zero-valent iron (ZVI) with an EMF generator (EMFG) to induce heat for chloride treatment in ground water^34^. However, using of EMF in geopolymers has not been reported. Therefore, this concept could be applied to a new GP heat curing procedure. Hence, the effect of WIP incorporated in GP must be investigated.

The use of iron-rich FA in GP resulted in a positive effect on GP properties^35^. In GP synthesis, the ferro-silico-aluminate GP could be formed using the high iron content in the prime material, in which some Al is substituted by Fe^36,37^. However, this process only occurred around 600–800 °C. Previous research on applying WIP in FA GP used conventional heat curing and revealed that the harmful actions of ferrous (Fe^2+^) compounds could block the geopolymerization and hinder the strength development of GP with time^36,38^. Even though the FA GP mixed with WIP resulted in unsatisfactory mechanical properties, applying EMF with WIP to GP is still of interest, and it has not been explored further.

Thus, this study explores the potential of electromagnetic induction in GP curing by applying WIP as a conducting material and investigating the physical and mechanical properties of the products. The energy consumption was compared with the conventional heated curing method. This EMF heat curing could play an important role in GP manufacture to enhance the strength quickly and improve the application of GP in construction.

## Experimental

### Materials

**Figure 1:**
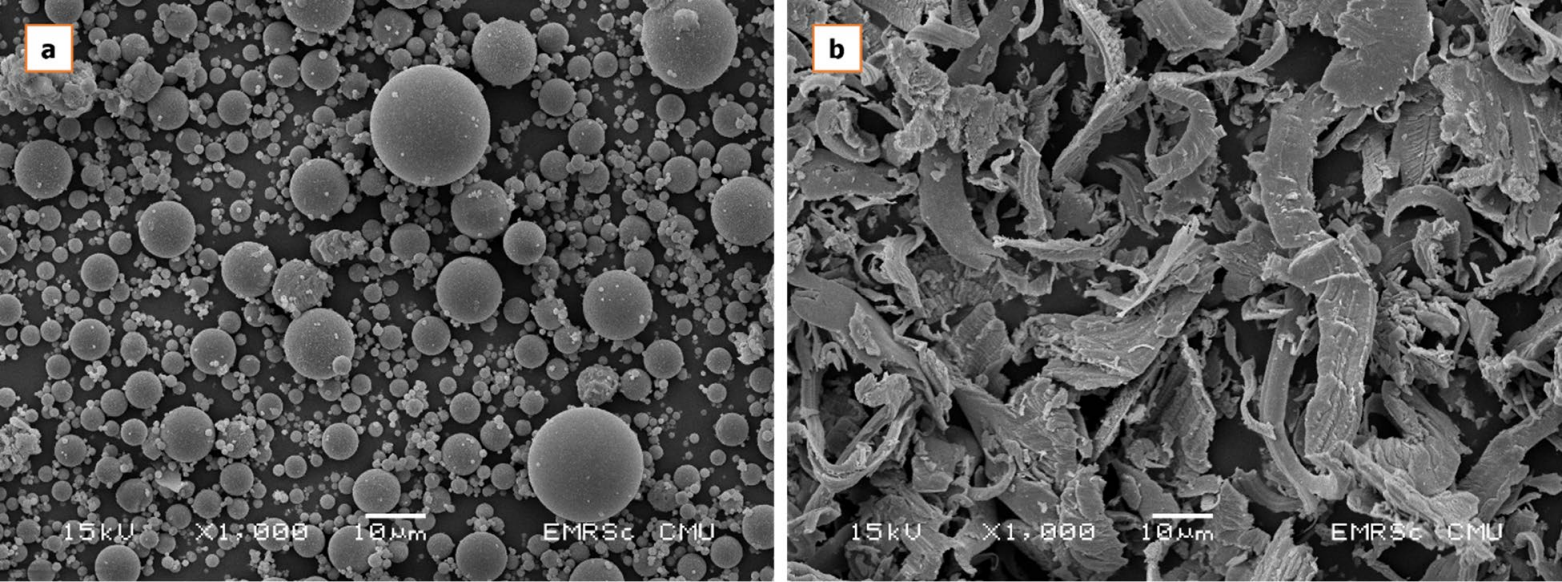
SEM microphotographs of (**a**) FA and (**b**) WIP at 1000 × magnification.

FA was obtained from Mae Moh coal-fired power plant, Lampang, Thailand. It was classified as Class C FA according to ASTM C618-11^39^. The properties of this FA are shown in Table 1. The high contents of CaO and iron oxide (Fe_2_O_3_) were detected by energy-dispersive X-ray fluorescence (EDXRF), using a JSX3400R (JEOL Ltd., Tokyo, Japan) and the FA has a reddish-brown color. The calcium content of this lignite coal is high and increased with the depth of mining^24^. Scanning electron microscopy (SEM) images of FA are shown in Fig. 1.

**Table 1:** Properties and chemical compositions of materials.

Chemical compositions	Fly Ash	WIP
Al_2_O_3_	8.52	–
SiO_2_	18.17	1.96
Fe_2_O_3_	29.85	95.95
CaO	31.41	–
SO_3_	8.43	–
K_2_O	2.47	–
TiO_2_	0.58	–
SrO	0.29	–
MnO	0.28	0.53
Cr_2_O_3_	–	1.56
Median particle size (D_50_), microns	19	22
Specific gravity	2.3	7.2

WIP was waste obtained from a bearing manufacturer in Thailand. The contaminated WIP was treated by using a detergent before the experiment. Its properties and chemical composition are also tabulated in Table 1. By EDXRF analysis, Fe_2_O_3_ was the main component at 95 wt% The SEM of the raw WIP is also shown in Fig. 1.

The alkali activator was a blended solution of sodium hydroxide (SH, NaOH) and sodium silicate (SS, Na_2_SiO_3_). Laboratory-grade SH was used (ACI Labscan Ltd., Thailand). To prepare 8 M NaOH, SH pellets were dissolved in distilled water and left overnight to obtain the solution at room temperature. A commercial-grade SS was purchased from a local distributor (Kusawad Chemical Group Ltd., Chiang Mai, Thailand) with the concentrations of 27 wt% SiO_2_ and 8 wt% Na_2_O. The alkali activator was the mixed solution of Na_2_SiO_3_ and NaOH with a weight ratio (SS/SH) of 1.00^40^.

### Geopolymer preparation

Firstly, the FA and WIP were oven-dried for 24 h and left to cool down at room temperature (25 °C). Then, FA was combined with the alkaline activator using a blending machine. A liquid and binder (L/B) mass ratio of 0.45 (L/B) was used^19^. The control GP paste (CGP) was produced as follows: the fresh GP paste was poured into a 40 × 40 × 160 mm^3^ prismatic mold and placed on the vibration table to eliminate the air bubbles. After that, the sample was left for 1 h to initiate hardening and then demolded. The samples were wrapped in plastic film to prevent moisture from leaking before the heat curing process was applied. Since a curing temperature of 45–80 °C could provide sufficient heat for the geopolymerization reaction^15,35^, a curing temperature greater than 80 °C could negatively affect the GP properties due to the sudden loss of moisture, resulting in the occurrence of micro-cavities in the GP structure^41^. Hence, the hot air oven was set up to cure CGP at 60 °C for 24 h. After curing, the samples were left to cool down to room temperature and then left to reach their testing ages of 3, 7, and 28 days. The mechanical properties, that is, the compressive and flexural strengths, were measured in accordance with BS-EN 196-1^42^.

For another group of samples, WIP contents of 5%, 10%, 15%, and 20% were mixed with fly ash by %weight of binder (FA) to produce the GPs for EMF curing, called the electromagnetically induced GP paste (EIGP) samples, where "EIGPx" indicates WIP-mixed GP paste, where x is the mass percentage of WIP. The preparation process was similar to CGP except for the heat curing method. EIGP samples were thermally cured using the EMF curing process. The detail of mix proportion is shown in Table 2.

**Table 2:** Mix proportions of GP pastes.

Mixture	FA (g)	WIP (g)	Alkali activator (g)	L/B	Testing age (days)	Heat curing method
CGP	100	0	45	0.45	3, 7, 28	Oven
EIGP5	100	5	45	0.45	3, 7, 28	EMFG
EIGP10	100	10	45	0.45	3, 7, 28	EMFG
EIGP15	100	15	45	0.45	3, 7, 28	EMFG
EIGP20	100	20	45	0.45	3, 7, 28	EMFG

### EMF curing process

**Figure 2:**
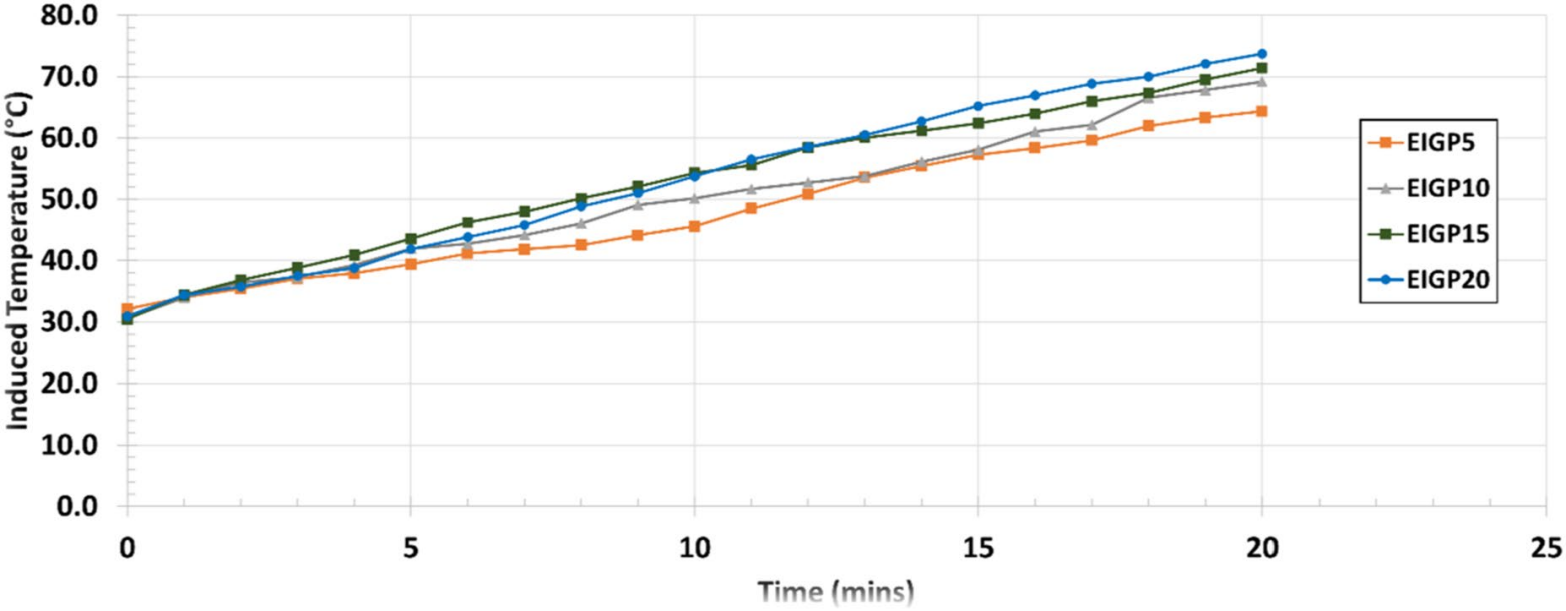
Induced heating temperature using EMF curing method.

**Figure 3:**
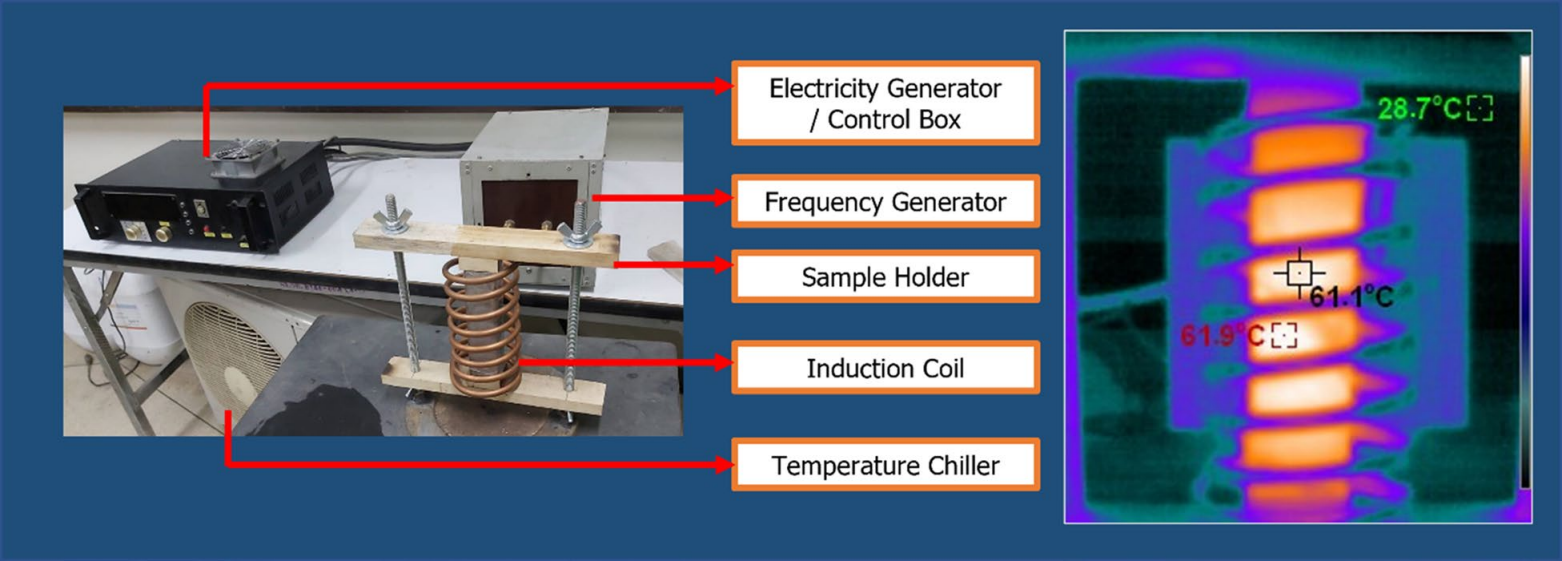
EMF equipment and temperature monitoring using a thermal imaging camera.

The custom-made EMFG was employed with reference to the 60 °C oven curing temperature. It should be noted that this EMFG used in the study cannot be automatically controlled to exactly obtain a target temperature. The samples were installed in the sample holder, leaving a 1 cm distance between the sample and the induction coil. To achieve the temperature of approximately 60 °C, the power was set to 1.18 kW and the frequency to 150–200 kHz. The temperature profiles were monitored using a thermal imaging camera (PONPE190, Protraction Intertrade Co., Ltd, Thailand). Figure 2 shows the results of heat induction. A heat curing duration of around 15 min could reach 55–65 °C for all of the EIGP samples. Hence, 15 min at these settings was selected for the EMF curing process. The EMF heat curing process details are shown in Table 3, and the EMF curing equipment and temperature monitoring are shown in Fig. 3.

**Table 3:** Details of oven and EMF curing processes.

Name	FA: WIP	Heat curing method	Frequency (kHz)	Power (kW)	Duration (mins)	Curing temperature (°C)
CGP (control)	100:0	Oven	–	4.20	3600	60
EIGP5	100:5	EMFG	150–200	1.18	15	55–65
EIGP10	100:10	EMFG	150–200	1.18	15	55–65
EIGP15	100:15	EMFG	150–200	1.18	15	55–65
EIGP20	100:20	EMFG	150–200	1.18	15	55–65

### Physical and mechanical testing

The bulk density of hardened GPs was measured prior to the mechanical properties test. The mechanical properties of hardened CGP and EIGP samples, that is, compressive strength and flexural strength, were measured for the 3, 7, and 28 day-aged samples using a CST compression machine (CST Instruments, Part Ltd., Thailand) following the BS-EN 196-1 standard. First, the flexural strength test was conducted on 40 × 40 × 160 mm^3^ prismatic samples using the three-point loading method with a 100-mm span length of two supporting points. Second, the parts obtained from the flexural strength test were then used for the compressive strength test at a loading rate of 1.00 mm/min and a 4 × 4 cm^2^ compressive area.

### Microstructure analysis

The X-ray diffraction (XRD) technique (Rigaku SmartLab, Rigaku Corporation., Tokyo, Japan) was used to investigate the morphology of materials. The EDX-SEM technique (JSM-5910LV, JEOL Ltd., Tokyo, Japan) was used to observe the microstructure and to identify the chemical compositions of products.

## Results and discussion

### Density

**Figure 4:**
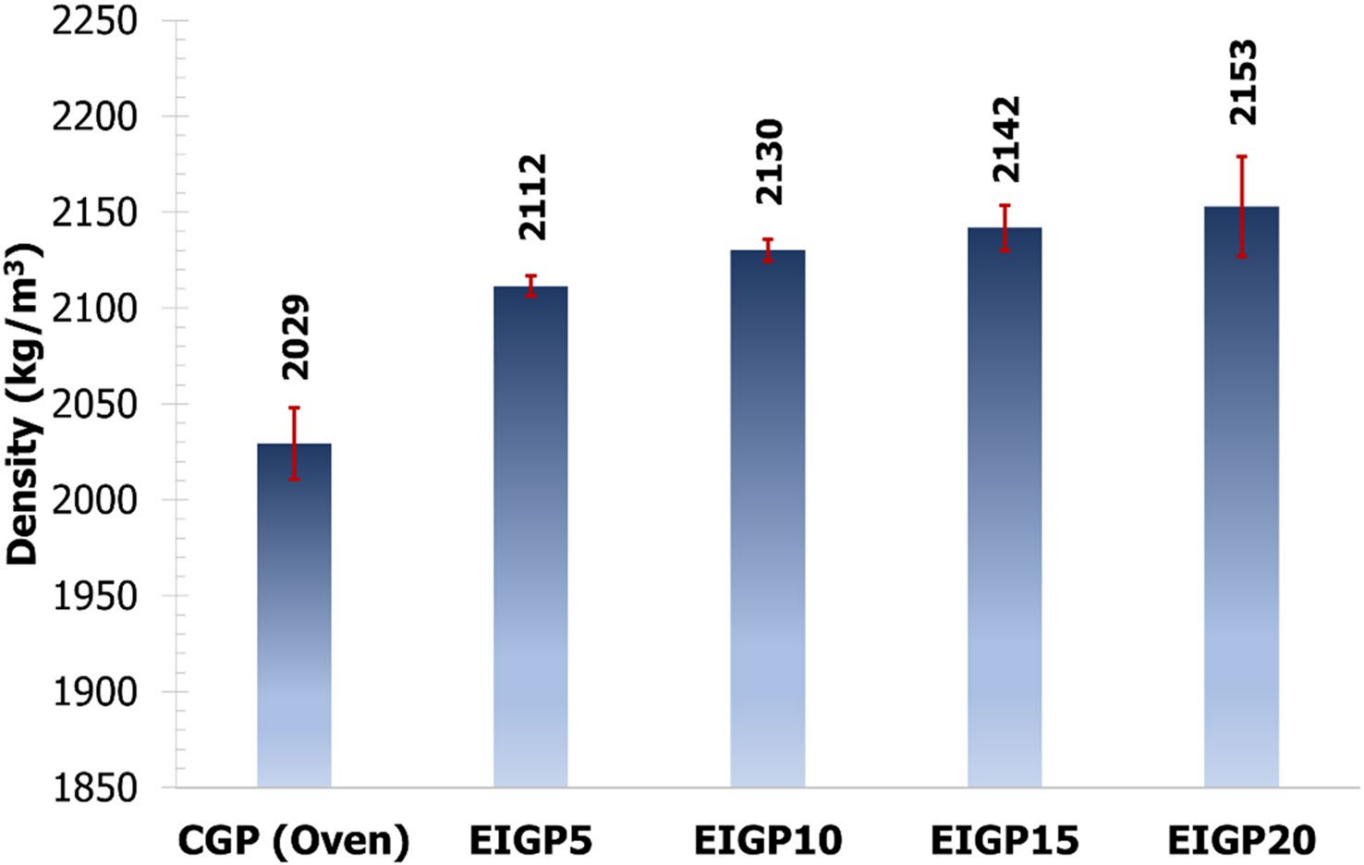
The densities of CGP and EIGPx samples at 28 days.

The bulk densities of 28-day-old hardened GP are shown in Fig. 4. The increase in the WIP content of the sample resulted in an increased GP density due to the difference between the specific gravity of WIP (7.2) and FA (2.3).

### Mechanical properties

**Figure 5:**
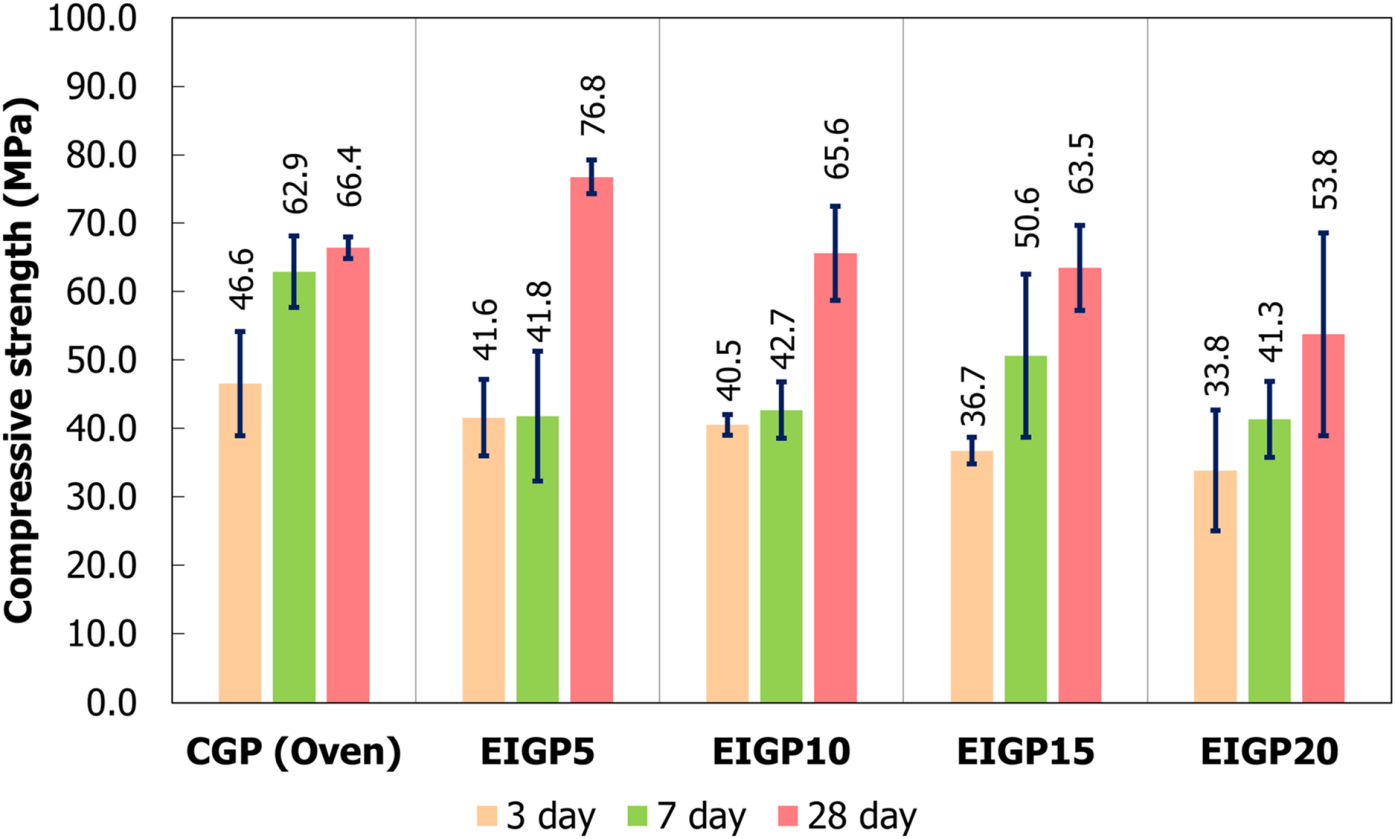
The compressive strengths of EIGP samples.

Figure 5 illustrates the compressive strength of the CGP and EIGP samples. The CGP samples gave a high early strength at 3 days of 46.6 MPa. At 7 and 28 days, the compressive strengths increased to 62.9 and 66.4 MPa, respectively. The strength developed with time owing to the hydration reaction from the high CaO content of the FA. The early strength of the EIGP samples was 41.6 MPa, slightly lower than that of CGP. Moreover, increased WIP content in FA GP led to lower early strength. For example, EIGP5 had a compressive strength of 41.6 MPa; simultaneously, EIGP20 had a strength of 33.8 MPa after 3 days of curing time. The decrease in compressive strength with increasing WIP content was due to the impurity in WIP and its particle shape^38^.

At 7 days, all the EIGP samples had greater compressive strength than the 3-day-old samples. Furthermore, the compressive strength of EIGP5 reached 76.8 MPa at 28 days, which is noticeably higher than the CGP (66.4 MPa). From the previous research, oven-cured WIP/FA GP could not develop this strength with time due to the formation of Fe compounds, the products of the reaction between the Fe and the alkaline solution (sodium tetrahydroxoferrate (II), Na_2_[Fe(OH)_4_]) in a microstructure^38^. The chemical reactions are shown in Eqs. 1 and 2. This Fe compound led to the volumetric expansion, causing stress and cracks in the GP matrix with longer curing times^43–45^. However, EMF heat-cured GP accomplished the strength development with time.

1 $$ {\text{Fe}}\left( {\text{s}} \right) + {\text{2NaOH}}\left( {{\text{aq}}} \right) \to {\text{Fe}}\left( {{\text{OH}}} \right)_{{2}} \left( {\text{s}} \right) + {\text{2Na}}^{ + } \left( {{\text{aq}}} \right) $$

2 $$ {\text{Fe}}\left( {{\text{OH}}} \right)_{{2}} \left( {\text{s}} \right) + {\text{2NaOH}}\left( {{\text{aq}}} \right) \to {\text{Na}}_{{2}} \left[ {{\text{Fe}}\left( {{\text{OH}}} \right)_{{4}} } \right]\left( {\text{s}} \right) $$

The EMF curing process positively affected geopolymerization and the hydration reaction due to the high calcium content of the FA. Generally, the heat evolution in GP depends on the heat energy that penetrates through a surface and transfers to the core by thermal convection. However, a gentle thermal gradient of the material slows down the rate of geopolymerization^31^ This may lead to less integrity of the geopolymeric gel formed. In contrast, the EMF-induced iron particles, which are a ferromagnetic material^46^, generate heat through the entire sample mass by the hysteresis loss phenomenon. The rapid temperature increase occurred throughout the matrix of the sample simultaneously. This situation could be theoretically described using the microwave radiation heat curing concept^25^. Previous research has reported that rapid heat evolution led to the greater dissolution of Al^3+^ and Si^4+^ at the surface of the FA particles, resulting in the increased integrity of the geopolymerization. In addition, the fast heating hindered the formation of Na_2_[Fe(OH)_4_], which is a main product for reduction in strength development. The presence of water molecules in the GP matrix was also concerning. Microwave heating directly vibrated the water molecules into H^+^ and OH^−^ ions. This dissociation of water molecules produced heat, but it decreased the water content available for later hydration reactions, including too-high curing temperatures, harming the GP structure. Curing at the boiling temperature of water resulted in rapid water evaporation, high pressure, and subsequent cracks in sample^25,41^. Therefore, microwave-cured GP could not attain great strength over a longer curing time because of the lack of water molecules for needed hydration reactions. Conversely, due to the induction, the EMF power vibrates only the WIPs to generate heat of around 50–70 °C in samples. Consequently, the water molecules were still present to react with CaO and form more hydration products (e.g., [N, C]–A–S–H gel).

**Figure 6:**
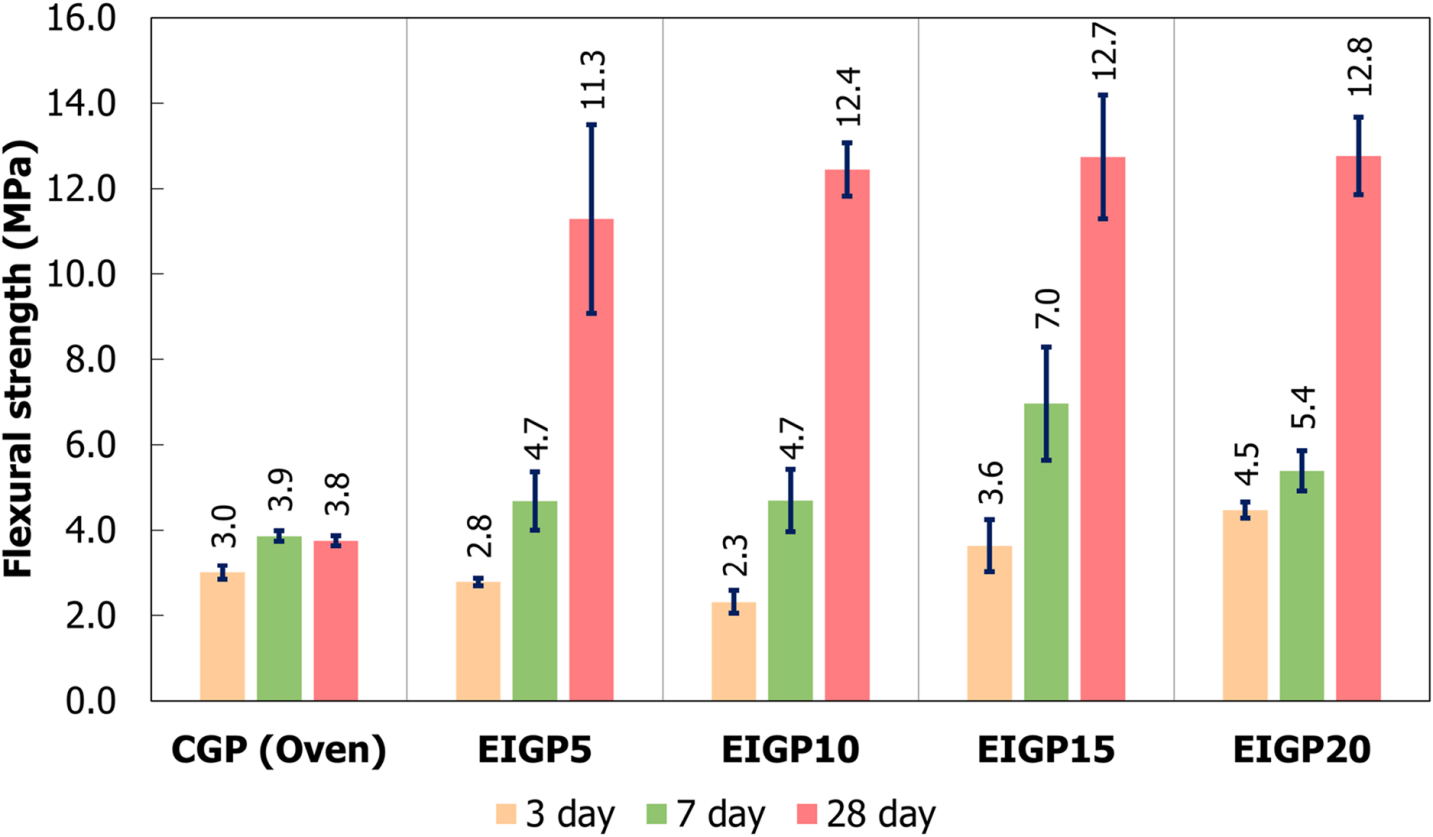
The flexural strength of CGP and EIGP samples.

The results of the flexural strength are presented in Fig. 6. The flexural strengths of CGP were 3.00, 3.86, and 3.80 MPa at 3, 7, and 28 days of curing, respectively. All samples, including CGP and EIGP, showed strength development. However, the EIGP samples had much greater flexural strength than the CGP. Although the 3-day-old samples of EIGP had the same flexural strength as the control sample, the flexural strength of the EIGP samples increased more than 10 MPa after 28 days of curing in every sample. The highest flexural strength was 12.8 MPa for EIGP20 after 28 days. This result was similar to previous research, which showed that the greater the content of WIP, the more flexural strength was obtained because the clusters of iron particles enhanced the flexural strength^38^. Hence, the addition of WIP not only increased the compressive strength but also greatly increased the flexural resistance in FA GP. In addition, WIP had a fibrous, flat-twisted shape with 10–30 microns in length, as shown in Fig. 1. This WIP could reinforce the intergranular space of GP and increases the flexural strength compared with that of the control.

### Microstructure analysis

**Figure 7:**
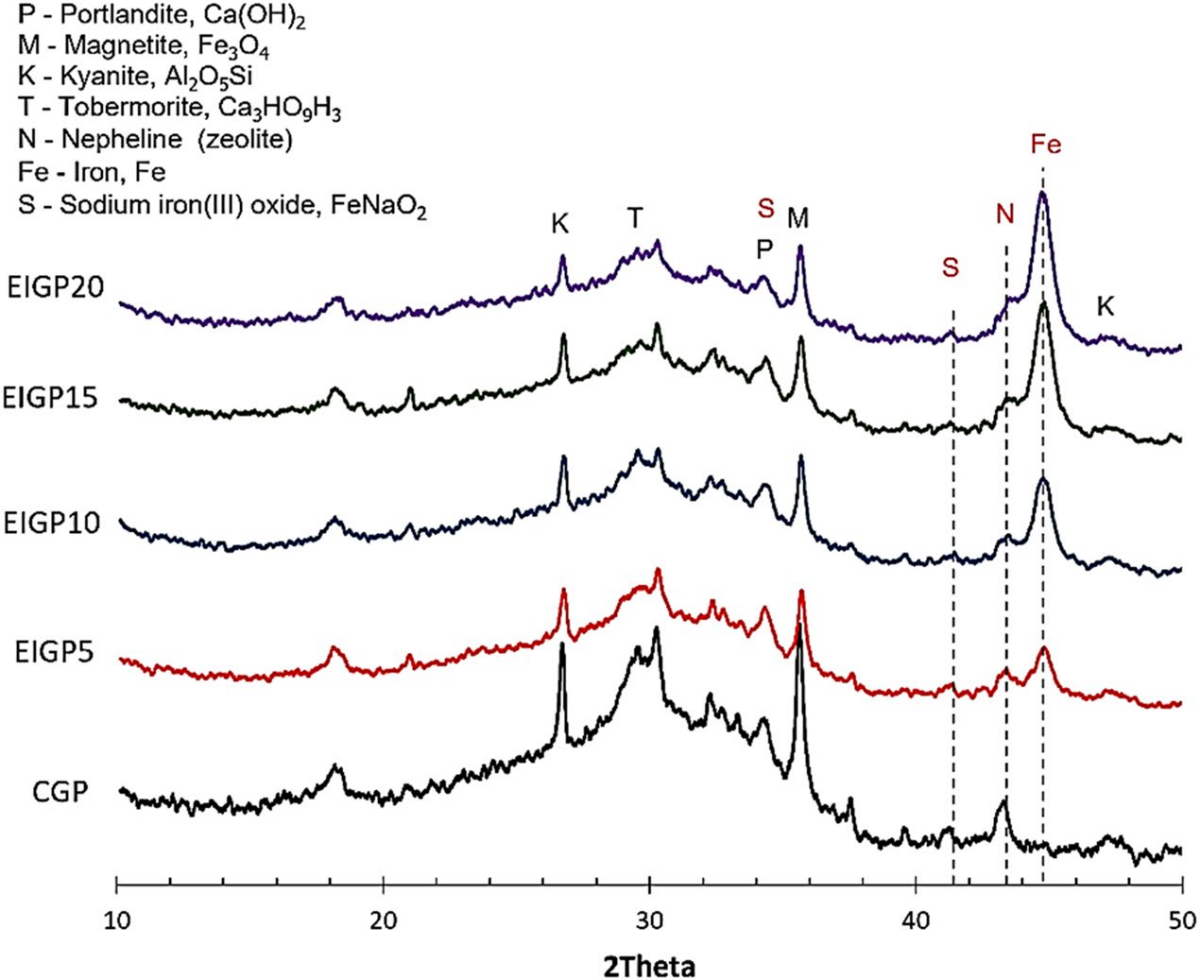
XRD results of EIGP samples.

All the samples were analyzed by XRD after 28 days of curing. XRD spectra of CGP and EIGP are shown in Fig. 7. The broad hump at the range of 20°–36° 2θ shows the major amorphous phase in GPs. It consisted of kyanite (Al_2_O_5_Si), tobermorite (Ca_3_HO_9_Si_3_), portlandite (Ca(OH)_2_), and magnetite (Fe_3_O_4_) for all samples. The difference between CGP and EIGP was between 2θ of 42°–46°, the phases of iron (Fe) and nepheline (zeolite). Due to the reaction between the WIP and the alkaline solution, sodium iron (III) oxide was detected in all samples. It was clear that the addition of WIP increased the peak intensity of the Fe and nepheline phases and reduced the intensity of the phases in the area of the broad hump (at 2θ = 20°–36°).

**Figure 8:**
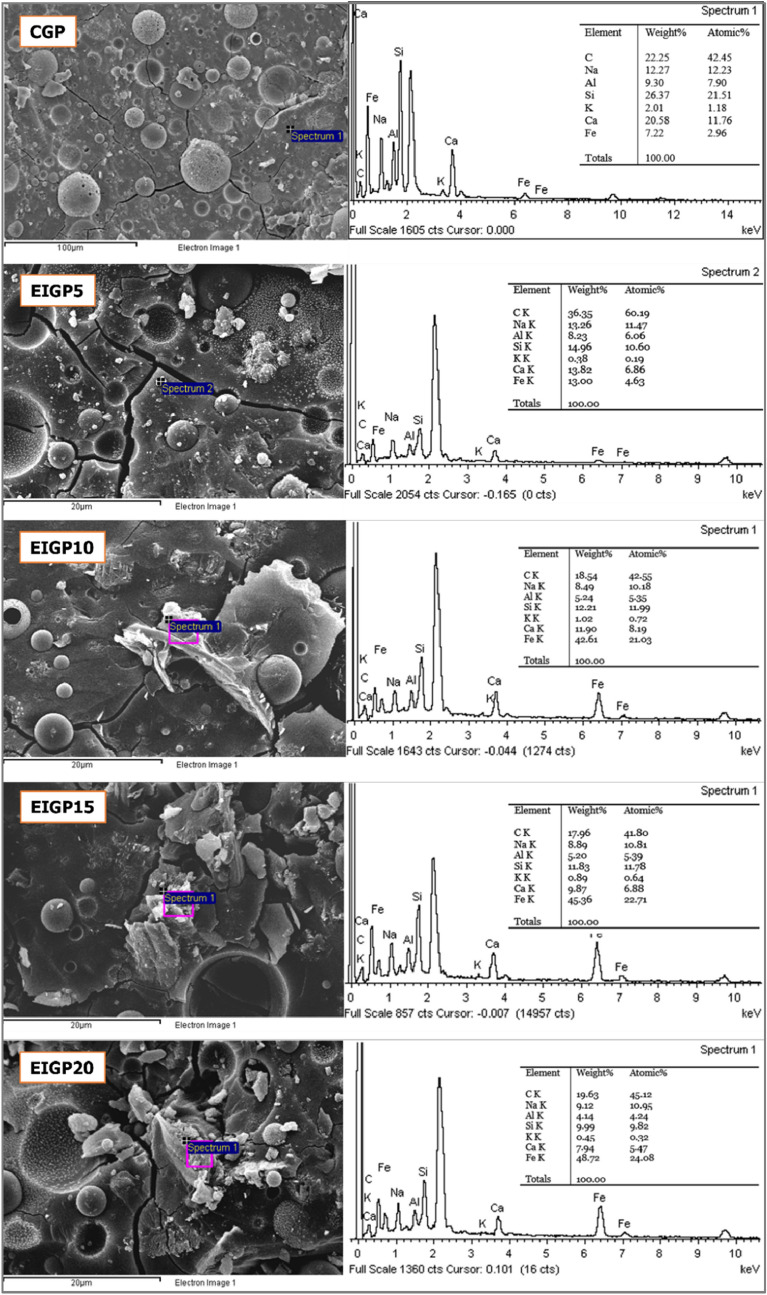
Detailed EDX spectral analysis of CGP and EIGP samples.

SEM–EDX techniques were used to study the microstructure of the CGP and EIGP samples, as seen in Fig. 8. Figure 8 shows the amorphous structure detected in CGP. Moreover, the unreacted FA particles were surrounded by geopolymeric gel, and the residual shells of FA after the reaction were seen in the GP matrix. The EDX spectra indicated the high Si, Al, and Ca contents, and the geopolymeric reaction and hydration products. EIGP samples appear similar to CGP, but the higher amounts of Fe due to the addition of WIP were detected. The flat and twisted shapes of the iron particles could be covered with C–S–H gel, and each particle was connected with geopolymeric gel, forming a cluster of iron particles. The geopolymer and C–S–H products of kyanite and tobermorite were found in composites detected by the XRD pattern (Fig. 7). These products had a lower density than WIP and formed over the WIP particles, as shown in the EDX analysis of Fig. 8. The products acted as a binder and led to the agglomeration of WIP particles. These WIP clusters were scattered around the GP matrix, enhancing the flexural resistance in EIGP samples.

From the EDX data, the Si/Al ratio of CGP was 2.84, whereas EIGP5, EIGP10, EIGP15, and EIGP20 had Si/Al ratios of 1.82, 2.33, 2.28, and 2.41, respectively. It has been reported that a Si/Al ratio in the range of 1.56–2.14 possibly results in fully condensed GP^11^. This ratio provided the dense matrix and high durability of GP. Since the Si/Al ratio affects the properties of geopolymers, the optimum Si/Al ratio provides microstructural stabilization and resistance to syneresis. This result improves mechanical properties^8^, as shown in compressive and flexural strengths (Figs. 5 and 6). A higher Si/Al ratio means high content of Si and a large quantity of unreacted fly ash due to a low amount of alkaline solution for reaction.

**Figure 9:**
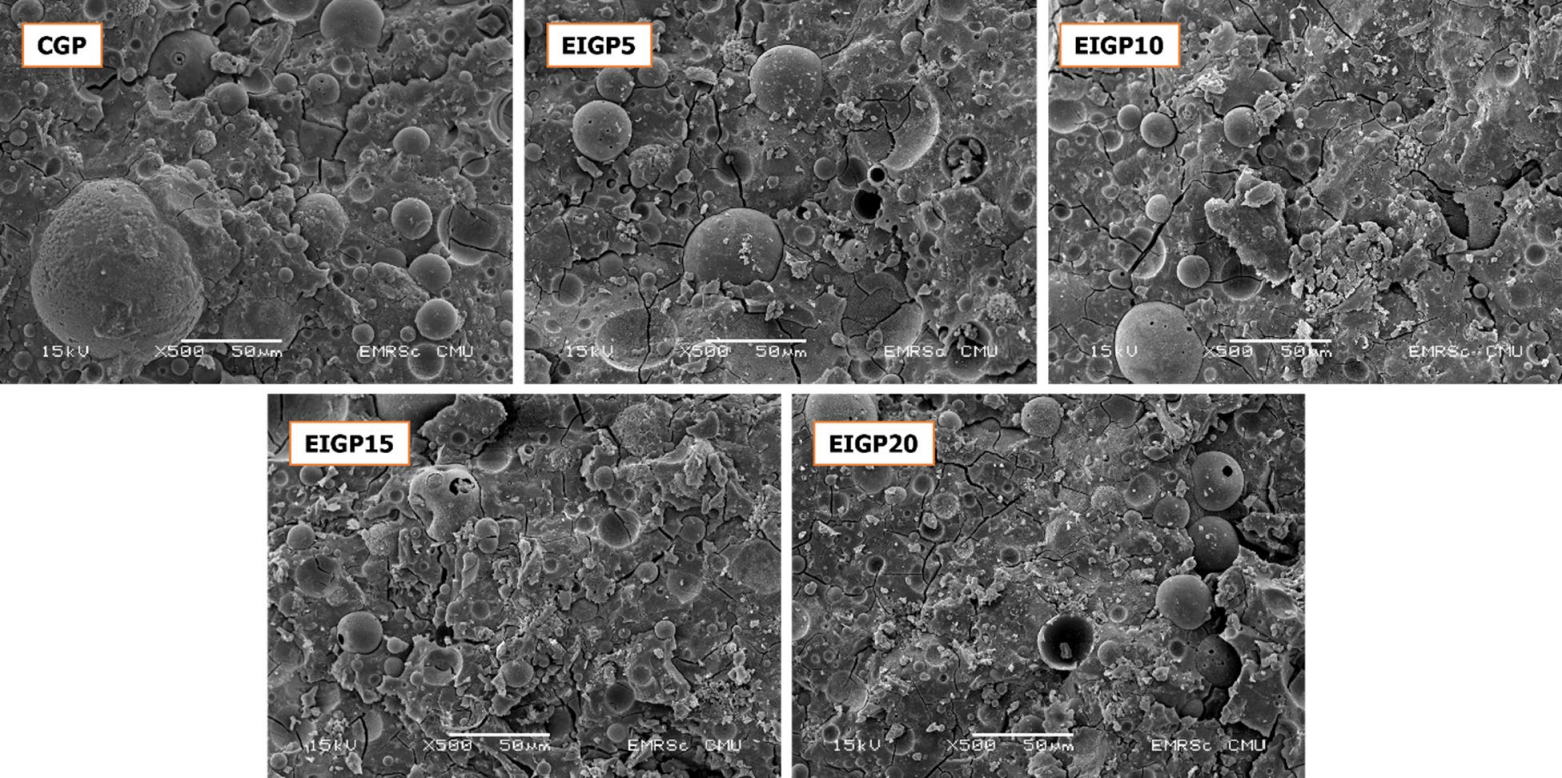
The SEM images of CGP and EIGP samples after 28 days of curing at 500 × magnification.

From the SEM images in Fig. 9, EIGP samples had denser structures than the CGP after 28 days of curing. The clusters of iron particles were detected throughout the GP matrix. According to the XRD analysis, the peak intensities of nepheline and the Fe phases increased with higher WIP content. It could explain that the occurrence of Na_2_[Fe(OH)_4_] and zeolite weakened the structure of GP compared with the other EIGP samples. Nevertheless, EIGP20 still had greater compressive strength than CGP because of the integrity of geopolymerization attained by the EMF curing process. EIGP5 was the best sample for the use of EMF in the heat curing process. Denser and stronger structures were formed by this method of GP manufacturing.

### Energy consumption

Table 4 shows the energy consumption of the curing process of the conventional method and the EMF method for the production of GP. The new alternative way of heat curing using EMF consumed less energy than the oven: a 99.70% reduction in energy consumption was recorded. Consequently, using the EMF method for heat curing not only increased the strengths of the GP but also dramatically reduced energy consumption in GP manufacturing.

**Table 4:** Details of energy consumption of EMF and conventional heat curing methods.

Heat curing method	Power (kW)	Duration (hour)	Energy consumption Unit (kW-h)	Energy consumption reduction (Oven − (EMF/Oven)) (%)
Conventional (Oven)	4.20	24	100.8	99.70
EMFG	1.18	0.25	0.295	

## Conclusions

In this study, the electromagnetic induction concept effectively enhanced the process of heat curing in GP. EMF curing greatly reduced the heat curing duration and energy consumption. A WIP content of 5% in GP cured by 1.18 kW EMFG for 15 min produced the highest compressive (76.8 MPa) and flexural strengths (11.3 MPa) after 28 days of curing, compared with those of the CGP (66.4 MPa and 3.8 MPa, respectively). The heat induction enhanced the integrity of the geopolymerization since the electromagnetic field rapidly promoted the dissolution of Si and Al ions from the surface of the FA to react with the alkali activator. Furthermore, EMF curing for 15 min reached 55–65 °C in the sample, which evaporated less water than the conventional heat curing method. Since the conventional heat curing method in the oven requires a long curing time, e.g., 24–48 h., to transfer the heat from the geopolymer's surface to the core of the sample, a more considerable amount of water is lost than the EMF method through evaporation. For EMF curing, samples could reach 55–65 °C in 15 min, and the heating covers the entire section of the sample due to the heat induction process, leading to the rapidly geopolymerization formation.

Therefore, the presence of moisture in the sample enhanced the reaction of the GP with extended curing time. In conclusion, the EMF curing process showed impressive results for contribution to the manufacture of FA GP.

## Data Availability

The datasets generated during and/or analyzed during the current study are available from the corresponding author on reasonable request.

## References

[1] Kumlai S, Jitsangiam P, Pichayapan P (2017). The implications of increasing temperature due to climate change for asphalt concrete performance and pavement design. KSCE J. Civ. Eng..

[2] Chindaprasirt P, Rattanasak U (2019). Characterization of the porous alkali-activated fly ash composite as solid adsorbent. Int. J. Greenhouse Gas Control.

[3] Chindaprasirt P, Rattanasak U (2020). Fabrication of self-cleaning fly ash/polytetrafluoroethylene material for cement mortar spray-coating. J. Clean. Prod..

[4] Jitsangiam P, Nikraz H, Siripun K (2009). Construction and demolition (C&D) waste as a road base material for western australia roads. Aust. Geomech. J..

[5] Jitsangiam P, Nikraz H (2013). Coarse bauxite residue for roadway construction materials. Int. J. Pavement Eng..

[6] Peerapong J, Boonserm K, Phenrat T, Chummuneerat S, Chindaprasirt P, Nikraz H (2015). Recycled concrete aggregates in Roadways: Laboratory examination of self-cementing characteristics. J. Mater. Civ. Eng..

[7] Huan Y, Siripun K, Jitsangiam P, Nikraz H (2010). A preliminary study on foamed bitumen stabilisation for Western Australian pavements. Sci. Res. Essays.

[8] Sounthararajah A, Bui H, Nguyen N, Jitsangiam P, Kodikara J (2018). Early-age fatigue damage assessment of cement-treated bases under repetitive heavy traffic loading. J. Mater. Civil Eng..

[9] Chindaprasirt P, Rattanasak U (2020). Synthesis of porous alkali-activated materials for high-acidic wastewater treatment. J. Water Process Eng..

[10] Davidovits J (1991). Geopolymer: Inorganic polymeric new materials. J. Therm. Anal..

[11] Davidovits, J. *Geopolymer: Chemistry and Application*. (Institut Geopolymere, Saint-Quantin 2008).

[12] Palomo A, Grutzeck MW, Blanco MT (1999). Alkali-activated fly ashes: A cement for the future. Cem. Concr. Res..

[13] Chindaprasirt P, Jaturapitakkul C, Chalee W, Rattanasak U (2009). Comparative study on the characteristics of fly ash and bottom ash geopolymers. Waste Manag..

[14] Chindaprasirt P, Jenjirapanya S, Rattanasak U (2014). Characterizations of FBC/PCC fly ash geopolymeric composites. Constr. Build. Mater..

[15] Chindaprasirt P, Rattanasak U, Vongvoradit P, Jenjirapanya S (2012). Thermal treatment and utilization of Al-rich waste in high calcium fly ash geopolymeric materials. Int. J. Miner. Metall. Mater..

[16] Cheng-Yong H, Yun-Ming L, Abdullah MMAB, Hussin K (2017). Thermal resistance variations of fly Ash geopolymers: Foaming responses. Sci. Rep..

[17] Maichin P, Suwan T, Jitsangiam P, Chindaprasirt P, Fan M (2020). Effect of self-treatment process on properties of natural fiber-reinforced geopolymer composites. Mater. Manuf. Process..

[18] Jitsangiam P, Suwan T, Pimraksa K, Sukontasukkul P, Chindaprasirt P (2019). Challenge of adopting relatively low strength and self-cured geopolymer for road construction application: A review and primary laboratory study. Int. J. Pavement Eng..

[19] Chindaprasirt P, Chareerat T, Sirivivatnanon V (2007). Workability and strength of coarse high calcium fly ash geopolymer. Cem. Concr. Compos..

[20] Suwan T, Fan M, Braimah N (2016). Internal heat liberation and strength development of self-cured geopolymers in ambient curing conditions. Constr. Build. Mater..

[21] Suwan T, Fan M (2014). Influence of OPC replacement and manufacturing procedures on the properties of self-cured geopolymer. Constr. Build. Mater..

[22] Kubba Z, Huseien FG, Sam ARM, Shah KW, Asaad MA, Ismail M, Tahir MM, Mirza J (2018). Impact of curing temperatures and alkaline activators on compressive strength and porosity of ternary blended geopolymer mortars. Case Stud. Constr. Mater..

[23] Somna K, Jaturapitakkul C, Kajitvichyanukul P, Chindaprasirt P (2011). NaOH-activated ground fly ash geopolymer cured at ambient temperature. Fuel.

[24] Chindaprasirt P, Rattanasak U (2017). Characterization of the high-calcium fly ash geopolymer mortar with hot-weather curing systems for sustainable application. Adv. Powder Technol..

[25] Chindaprasirt P, Rattanasak U, Taebuanhuad S (2013). Role of microwave radiation in curing the fly ash geopolymer. Adv. Powder Technol..

[26] Hong S, Kim H (2019). Effects of microwave energy on fast compressive strength development of coal bottom ash-based geopolymers. Sci. Rep..

[27] Suwan T, Paphawasit B, Fan M, Jitsangiam P, Chindaprasirt P (2021). Effect of microwave-assisted curing process on strength development and curing duration of cellular lightweight geopolymer mortar. Mater. Manuf. Process..

[28] Graytee A, Sanjayan JG, Nazari A (2018). Development of a high strength fly ash-based geopolymer in short time by using microwave curing. Ceram. Int..

[29] Shee-Ween O (2021). Cold-pressed fly ash geopolymers: Effect of formulation on mechanical and morphological characteristics. J. Market. Res..

[30] Ranjbar N, Kashefi A, Ye G, Mehrali M (2020). Effects of heat and pressure on hot-pressed geopolymer. Constr. Build. Mater..

[31] Suwan T, Fan M, Braimah N (2016). Micro-mechanisms and compressive strength of Geopolymer-Portland cementitious system under various curing temperatures. Mater. Chem. Phys..

[32] Center of Excellence on Hazardous Substance Management. 100 Types of Industrial Waste Quantities (2008–2011) in Thailand. *Chulalongkorn University* http://recycle.dpim.go.th/wastelist/download_files/G/waste_quantity.pdf (2012).

[33] Liu K, Fu C, Dai D, Li W, Li S, Xu X (2019). Induction heating performance of asphalt pavements incorporating electrically conductive and magnetically absorbing layers. Constr. Build. Mater..

[34] Phenrat T, Thongboot T, Lowry GV (2016). Electromagnetic induction of zero-valent iron (ZVI) powder and nanoscale zero-valent iron (NZVI) particles enhances dechlorination of trichloroethylene in contaminated groundwater and soil: Proof of concept. Environ. Sci. Technol..

[35] Kumar S, Yankwa DJN, Kumar A, Kumar S (2016). Geopolymerization behavior of fine iron-rich fraction of brown fly ash. J. Build. Eng..

[36] Davidovits J, Davidovits R (2020). Ferro-sialate geopolymers (-Fe-O-Si-O-Al-O-). Geopolym. Inst..

[37] Lemougna PN, MacKenzie KJD, Jameson GNL, Rahier H, Chinje MUF (2013). The role of iron in the formation of inorganic polymers (geopolymers) from volcanic ash: A 57Fe Mössbauer spectroscopy study. J. Mater. Sci..

[38] Nongnuang T, Jitsangiam P, Rattanasak U, Tangchirapat W, Suwan T, Thongmunee S (2021). Characteristics of waste iron powder as a fine filler in a high-calcium fly ash geopolymer. Materials.

[39] ASTM C618-19. *Standard Specification for Coal Fly Ash and Raw or Calcined Natural Pozzolan for Use in Concrete*. *ASTM International* (ASTM International, 2019). 10.1520/C0618-19.

[40] Rattanasak U, Chindaprasirt P (2009). Influence of NaOH solution on the synthesis of fly ash geopolymer. Miner. Eng..

[41] Bakria AMM (2011). The effect of curing temperature on physical and chemical properties of geopolymers. Phys. Procedia.

[42] BS EN 196-1. *Methods of testing cement. Determination of strength*. *British Standards Institution* (2016).

[43] Jaśniok T, Słomka-Słupik B, Zybura A (2014). The concrete reinforcement chloride corrosion, immediately after its initiation. Cem. Wapno Beton.

[44] Tang SW, Yao Y, Andrade C, Li ZJ (2015). Recent durability studies on concrete structure. Cem. Concr. Res..

[45] Zhao Y, Yu J, Hu B, Jin W (2012). Crack shape and rust distribution in corrosion-induced cracking concrete. Corros. Sci..

[46] Phenrat T, Saleh N, Sirk K, Tilton RD, Lowry GV (2007). Aggregation and sedimentation of aqueous nanoscale zero-valent iron dispersions. Environ. Sci. Technol..

